# An integrated soil-crop system model for water and nitrogen management in North China

**DOI:** 10.1038/srep25755

**Published:** 2016-05-16

**Authors:** Hao Liang, Kelin Hu, William D. Batchelor, Zhiming Qi, Baoguo Li

**Affiliations:** 1 College of Resources and Environmental Sciences, China Agricultural University, Key Laboratory of Arable Land Conservation (North China), Ministry of Agriculture, Beijing 100193, P.R. China; 2 Biosystems Engineering Department, Auburn University, Auburn, AL, 36849, USA; 3 Department of Bioresource Engineering, McGill University, Sanite-Anne-de-Bellevue, QC, H9X 3V9, Canada

## Abstract

An integrated model WHCNS (soil Water Heat Carbon Nitrogen Simulator) was developed to assess water and nitrogen (N) management in North China. It included five main modules: soil water, soil temperature, soil carbon (C), soil N, and crop growth. The model integrated some features of several widely used crop and soil models, and some modifications were made in order to apply the WHCNS model under the complex conditions of intensive cropping systems in North China. The WHCNS model was evaluated using an open access dataset from the European International Conference on Modeling Soil Water and N Dynamics. WHCNS gave better estimations of soil water and N dynamics, dry matter accumulation and N uptake than 14 other models. The model was tested against data from four experimental sites in North China under various soil, crop, climate, and management practices. Simulated soil water content, soil nitrate concentrations, crop dry matter, leaf area index and grain yields all agreed well with measured values. This study indicates that the WHCNS model can be used to analyze and evaluate the effects of various field management practices on crop yield, fate of N, and water and N use efficiencies in North China.

Irrigation and fertilization are the two major factors in obtaining high grain yields around the world. As one of the largest countries in the world, China has a shortage of water resources^1^ which may limit the ability of China to produce enough food for its population in the future. Agricultural water consumption accounts for more than 65% of total national water use under current agricultural management practices in China, however, irrigation water use efficiency (IWUE) is only about 40% due to open channel irrigation practices^2^. According to the National Bureau of Statistics of China^3^, the total nitrogen (N) fertilizer use was nearly 23.9 million tons (Mt) in 2013, which is about twice as much as in 1985, while the grain production increased from 378.6 Mt in 1985 to 601.9 Mt in 2013 (only 1.59 times). The increased rate of grain production is much lower than that of N fertilizer, illustrating that the N use efficiency (NUE) is very low and a large amount of N fertilizer is lost to the environment.

The North China Plain (NCP) is the most important wheat and maize production region in China, and it produces approximately 60–80% of China’s wheat and 35–40% of China’s maize each year^4^. However, the NCP contains only 8% of China’s total water resources^5^,^6^. The area is currently facing a severe limitation of water evidenced by a rapid drop in the aquifer that is used for irrigation^7^,^8^. It is also an area with increased agricultural non-point source pollution from N management practices^9^. Since the 1970s, the groundwater in NCP has experienced a long-term over-extraction, and areas of groundwater depression have formed, leading to a drop in the water table of approximately 1 m per year in some areas, which is unsustainable. Current farming management practices are degrading the geological environment and reducing groundwater resources^5^,^7^,^8^. A winter wheat/summer maize double cropping system is widely implemented by farmers in this region, with the majority of the irrigation used for the winter wheat crop. Farmers in NCP typically apply excess N fertilizer to achieve high grain yields, which has resulted in low N use efficiency (approximately 30%) in the cropping system, and residual ratio of nitrate in soil as high as 25–45%^10^. The low NUE has resulted in environmental problems such as groundwater nitrate pollution^11^,^12^, and surface water eutrophication^9^. In order to increase crop yields and improve WUE, NUE and reduce the risk of environmental pollution, modeling tools and analytical techniques are needed to quantify how cropping systems may respond to alternative best management practices.

Conventional field experiments play an important role in assessing the effects of single or multiple factors on crop yield and the fate of N. The processes of soil water dynamics and N cycling in soil-crop systems are complex due to variation in soil properties, weather, and environmental conditions. Models have been successfully used to analyze these complex systems under spatial and temporal variability. Crop and soil models have been used to optimize water and N management practices by simulating water and N dynamics, organic matter turnover and crop growth. Examples of models used around the world include WOFOST^13^, DAISY^14^, HERMES^15^, EPIC^16^, DNDC^17^, RZWQM^18^, DSSAT^19^, APSIM^20^, WNMM^21^, SPACSYS^22^, HYDRUS1D^23^.

Soil-crop system models are typically designed to answer specific questions, and incorporate methods to simulate processes to achieve their objectives^24^,^25^,^26^. For instance, The HYDRUS1D model has been widely used to simulate the movement of water, salt, nutrient and contaminants (pesticide, heavy metal and pathogenic microorganism, etc). The model simulates one-dimensional movement of water, heat and multiple solutes through a series of partial differential equations and kinetic equations. However, the model could not simulate crop growth and soil-plant interactions through the root system, which makes the HYDRUS1D model unsuitable to optimize fertilizer management in agricultural production. Recently, HYDRUS1D has been coupled with the WOFOST crop model and it was used to study irrigation management in semi-arid regions, but the carbon (C) and N processes were ignored in this research^27^. Wang *et al*.^28^ used HYDRUS1D to evaluate the effect of heavy rainfall and high-intensity irrigation rates on nitrate leaching in NCP, and recommend decreasing the individual irrigation amount and increasing the irrigation times after fertilizer application. However, this research failed to simulate the crop growth due to the lack of a crop module in HYDRUS1D. DAISY is a process-based cropping systems model that was initially focused on quantifying the manure (straw) effects on soil-crop systems under various management practices. It was coupled with MIKE SHE hydrological model to conduct regional analysis. However, the model is difficult for policy-makers and producers to use in the NCP because of its complex input and output file structure and lack of a user-friendly interface. The RZWQM model, coupled with the DSSAT crop models and the SHAW energy balance model has primarily been used to study crops grown under extensive management and climate in USA. Hu *et al*.^29^ adopted RZWQM model to evaluate water and N management in a double cropping system in the NCP, and found that the application rates of water and N could be reduced by about half. However, this model overlooks the intensive management factors such as high planting density, mulching, and other management factors. The DNDC model was originally designed to simulate soil C and N dynamics and trace gas emissions. Li *et al*.^30^ adopted the DNDC model to simulate the impacts of alternative management practices on greenhouse gas (GHG) emission in the NCP, and suggested that manure application or crop residue incorporation rather than increasing N fertilizer application rate would more efficiently mitigate GHG emissions from the cropping system. Because crop growth in the DNDC model is estimated using a generalized crop growth curve, it is not capable of simulating the effects of climate on crop growth and its interactions with soil biogeochemical processes. WOFOST and DSSAT represent plant growth process very well and are well structured for evaluating management practices on crop growth and evaluating sustainability of cropping systems. For example, Chen *et al*.^31^ studied the effects of climate variability and water management on crop water productivity using the WOFOST model in the NCP, and recommending irrigation strategies for wheat and maize. However, these models are more limited in their representation of soil processes. Moreover, some models, such as DSSAT, EPIC, and APSIM, were developed using the FORTRAN language. Most models still rely heavily on their legacy code. This reliance originates from significant past efforts to build model components, but this also limits the evolution of the code toward more modern Windows and internet based implementation^32^.

The intensive wheat-maize double-cropping system used in the NCP is characterized by conservation tillage, variable crop variety, planting date, planting density, mulching, and other management practices. Farmers typically use excess water and N fertilizer to insure high yields^33^,^34^, which drives the development of a new model to aid the field management in the NCP. Thus, the aims of this study were to (i) develop an integrated crop and soil water, C and N management model WHCNS (soil Water Heat Carbon Nitrogen Simulator) based on components of existing and widely tested soil-crop system models, (ii) to compare the model performance with 14 other models based on the public available common datasets from European, to (iii) evaluate the model under different climate zones, soils, crop rotations, and water and fertilizer management practices in North China, and to (iv) develop a water and N management model using object-oriented program for better delivery on modern Windows platforms.

## Materials and Methods

### Model description

#### The framework of WHCNS model

**Figure 1:**
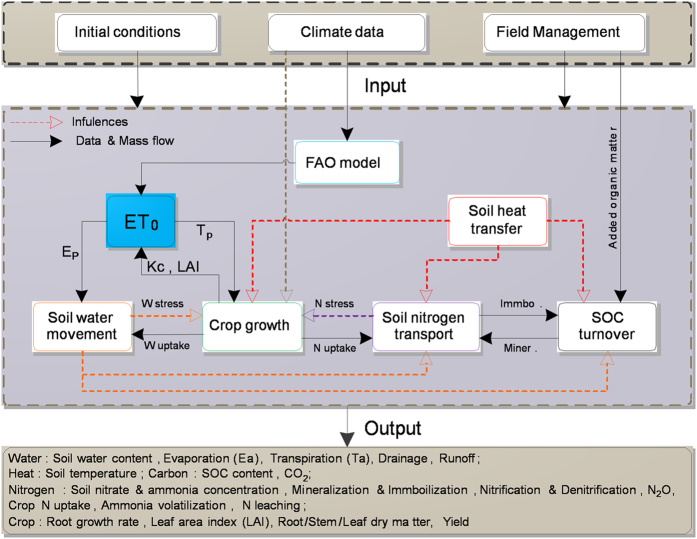
The conception framework of the WHCNS model.

The WHCNS (soil Water Heat Carbon Nitrogen Simulator) model consists of five main modules, including soil water, soil heat transfer, N transport, soil organic carbon (SOC) turnover and crop growth modules. The model was programmed in the C++ object-oriented programming language and the modules can be run together as a system or independently to study processes in isolation. The conceptual model is shown in Fig. 1.

#### Soil water dynamics

Surface water runoff is simulated for daily rainfall using the SCS curve number equation^35^. Subsequently, soil water infiltration is computed using the Green-Ampt model^36^. The process of soil water redistribution was incorporated into the model using the Richard’s equation as described by Šimůnek *et al*.^23^. The reference crop evapotranspiration ET_o_ is estimated using the Penman-Monteith equation^37^ solved using standard grass with an assumed height of 0.12 m and a surface resistance of 70 s m^−1^. The crop coefficient is used to calculate actual crop potential evaporation. And then, using leaf area index (LAI), separate potential crop transpiration and potential soil evaporation^38^.

#### Soil heat dynamics

The simulation of one-dimensional heat transfer was taken from the HYDRUS1D model, which is described with the convection-dispersion equation^23^. The top and bottom boundaries are set constant boundary conditions. The temperature of the top soil layer is estimated based on the daily air temperature and leaf area index^17^. The bottom boundary temperature is estimated used by the method used in the DNDC model^17^.

#### Soil solute transport

The transport of soil NH^+^_4_-N and NO^−^_3_-N was simulated using the convection-dispersion equation (CDE), and a generalized nonlinear adsorption isotherm was used to consider the adsorption-desorption process between the liquid and solid phase as described in HYDRUS1D model^23^. The WHCNS model assumes an equal crop absorption ratio of NH^+^_4_-N and NO^−^_3_-N. Each N transformation process was computed as a sink-source term in the CDE, and each of the processes are described detail in next two sections. The boundary conditions (Cauchy type) for the solute (NH^+^_4_-N and NO^−^_3_-N) transport equation was used to describe the solute flux at the upper or lower boundary. This CDE was solved by the general upwind difference method, and this procedure effectively avoids numerical dispersion and oscillation even under the conditions of dramatic changes in solute concentration without using dense nodes^39^. Surface broadcast and deep fertilizer applications are regarded as uniformly incorporated within the top 1 cm of soil or at the prescribed application depth (usually 5–10 cm) in the soil, respectively.

#### Soil organic carbon and N transformation

The module to simulate organic matter turnover was taken from the DAISY model^14^,^40^, which was originally used for quantitatively evaluating the effect of animal manure on soil-crop systems. The organic matter in soil is divided into three main pools, i.e. dead native soil organic matter (SOM), soil microbial biomass (SMB), and added organic matter (AOM). Each of these distinct pools were considered to contain a continuum of substrate qualities, but to facilitate the description of all turnover processes by first-order kinetic, each of these main pools were divided into two subpools: one with a slow turnover rate (e.g. SOM1, SMB1, and AOM1), and the other with a fast turnover rate (e.g. SOM2, SMB2, and AOM2). The decay rate was proportional to the size of the pool:


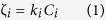


where ζ_*i*_ is decomposition or decay rate of pool *i* (kg C cm^−3^ d^−1^), *C*_*i*_ is carbon content in soil of pool *i* (kg C cm^−3^), and 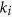 is decomposition or decay rate of pool *i* (d^−1^). The decomposition or decay rate at actual condition (*k*_*AOM*_) was derived as the rate at standard abiotic conditions multiplied by abiotic functions taking into account the effects of soil temperature, soil water content, and clay content of the soil. For pools of added organic matter (AOM1 and AOM2), the decomposition rate was calculated from Eq. (2):


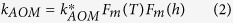


where 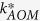 is the decomposition rate coefficient of AOM at standard conditions (d^−1^), *F*_*m*_(*T*) and *F*_*m*_(*h*) are temperature and pressure potential functions, respectively, as detailed by Hansen *et al*.^14^. For pools of dead native SOM (SOM1 and SOM2), the decomposition rate was computed by


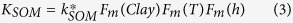


where 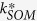 is decomposition rate coefficient of SOM at standard conditions (d^−1^), *F*_*m*_(*Clay*) is clay content function^41^. For pools of microbial biomass (SMB1 and SMB2), the decay rate at the standard conditions as specified in relation to Eq. (3) was assumed to include a specific death rate constant and a specific maintenance rate coefficient:


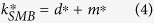



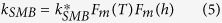


where 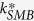 is decay rate coefficient of SMB at standard conditions (d^−1^), 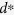 is death rate coefficient for SMB at standard condition (d^−1^), and 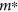 is the maintenance coefficient for SMB at standard condition (d^−1^). AOM can be transformed into SMB, and then SMB can be transformed into SOM, which can also be utilized by microorganisms and transformed into SMB. Hansen *et al*.^40^ assumed that soil greenhouse gas emissions and N mineralization-immobilization is closely related to soil microbial activity. Production of CO_2_ results from all C-fluxes into the microbial biomass (SMB) pools (substrate utilization efficiencies being less than unity). We assumed a linear relationship between potential denitrification rate and CO_2_ emission^42^. Net mineralization rate of N was calculated by the C/N ratio:


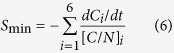


where *S*_*min*_ is net mineralization of SOM (ug cm^−3^ d^−1^), (*C/N*)_*i*_ is C/N ratio of pool *i* (AOM1, SOM and SMB).

#### Inorganic nitrogen transformation

##### Urea hydrolysis

The urea hydrolysis process was computed by a first-order reaction kinetic equation^21^:


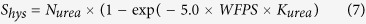


where *N*_*urea*_ is the urea-N concentration in soil (ug cm^−3^), *WFPS* is the fraction of water-filled pore space (−), and *K*_urea_ is the first order kinetic rate constant (d^−1^). Typically urea hydrolysis is completed in several days under hot conditions, while under cold conditions, it takes longer. The models NLEAP, GLEAM, EPIC and others assume urea hydrolysis occurs instantly, and even treated the urea as ammonium directly.

##### Ammonia volatilization

Ammonia volatilization was simulated using a method proposed by Freney *et al*.^43^:


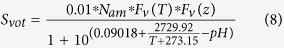


where *pH* is the value of pH, *N*_*am*_ is the concentration of ammonium N in soil (ug cm^−3^), F_v_(*T*) and F_v_(*z*) are soil temperature functions (°C) and soil depth functions (cm) proposed by Freney *et al*.^43^.

##### Nitrification

Nitrification was simulated by the Michaelis-Menten kinetic equation, and modified by soil moisture and temperature^14^,^40^:


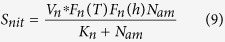


where *V*_n_* is the maximum nitrification rate at 10 °C under optimal soil water condition (ug cm^−3^ d^−1^),* K*_*n*_ is a half saturation constant (ug cm^−3^), *F*_*n*_(*T*) is the soil temperature function and *F*_*n*_(*h*) is the pressure potential function proposed by Flowers and O’Callaghan^44^ and Addiscott^45^, respectively.

##### Denitrification

According to Lind^42^, the potential denitrification rate (the extreme anoxic and ample nitrate supplement condition) of the soil can be expressed as a linear function of the CO_2_ evolution rate. The actual denitrification rate is determined either by the transport of nitrate to the anaerobic micro sites or the actual microbial activity at these sites. The increased tortuosity when the soil dries leads to discontinuity and thus, denitrification is very limited in dry soil. In the case of ample supply of nitrate, the actual denitrification rate was determined by multiplying the potential denitrification rate by a modified function. Hence, the actual denitrification process was simulated as:


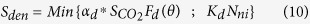


where Sden is rate of denitrification (ug cm-3 d-1), *α*_d_* is an empirical constant (default value 0.1 g Gas-N/g CO_2_-C); *S*_*CO*2_ is derived from the organic matter model as the evolution of CO_2_ from the decomposition of organic matter (ug cm^−3^ d^−1^); *K*_*d*_ is an empirical proportionality factor; *N*_*ni*_ is the concentration of nitrate N in soil (ug cm^−3^); *F*_*d*_(*θ*) is soil water content function described in DAISY model^40^.

#### Crop growth model

The simulation of crop growth and development stage, LAI, biomass accumulation and allocation, maintenance respiration and growth respiration was computed based on modifications of the PS123 model^46^, which is a generic dynamic crop model to simulate annual crop growth of many crops. The modifications are outlined below.

##### Crop emergence

The crop relative development stage was divided into two stages: sowing to emergence (stage1) and emergence to maturity (stage2). The first stage was described by a linear function of sowing depth, which was proposed by Mao^47^:


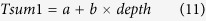


where *Tsum*1 is the heat requirement for stage1 (°C), *a* and *b* are empirical coefficients, and *depth* is sowing depth (cm).

##### Root growth

Root growth and development was computed by Šimůnek *et al*.^23^:


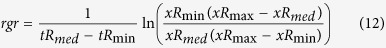



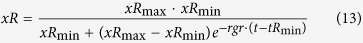


where *t* is time (d), *rgr* is root growth rate (cm d^−1^), *tR*_min_ is the initial root growth time (d), *tR*_*med*_, *xR*_*med*_ is the time and root depth at the midpoint of development, respectively (cm), *xR*_min_ and *xR*_*max*_is the initial and maximum rooting depth (cm), respectively, and *xR* is the root depth (cm) at time *t*. For overwintering crops, when the temperature in the winter is below zero for 5 continuous days, the roots stop growing. If the soil temperature exceeds 0 °C for 5 continuous days in the spring, the root system begins to grow again.

##### Root water and nitrogen uptake

Root growth and development was computed by a method proposed by Šimůnek *et al*.^23^, and root water uptake was calculated by:


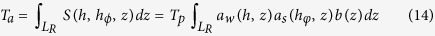


where *T*_*a*_ is actual root water uptake (or crop transpiration) (cm d^−1^), *L*_*R*_ is root depth (cm), *a*_*w*_(*h, z*) is a water stress response function^48^, *a*_*s*_(*h*_*φ*_, *z*) is a salinity stress response function^49^, and *b*(*z*) is a root distribution function^48^.

Šimůnek and Hopmans^50^ introduced a critical value of water stress index *ω*_*c*_, called the root adaptability factor. This is a threshold value above which root water uptake is reduced in water limited layers of the root zone but the plant compensates by uptaking more water from other layers that have sufficient available water. However, some reduction in potential transpiration occurs below this threshold value, though smaller than that for water uptake without compensation. The water stress index, *cf*(*w*), was calculated from Eq. (15).


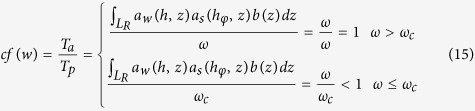


The shoot and root of crops have different N contents, and the actual root N uptake is determined by the minimum value between the N demand of the crop and soil supply. The crop N stress calibration factor, *cf*(*N*), was computed based on the CERES model^38^ by:


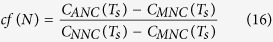


where *T*_*s*_ is the accumulated effective temperature from crop emergence (°C); *C*_*ANC*_, *C*_*NNC*_ and *C*_*MNC*_ are the actual crop N content (%), critical crop N content (%) and minimum crop N content (%), respectively.

### The characteristics of WHCNS model

The main characteristics of the integrated model includes:

- The primary modules were adapted from existing widely used soil-crop system models, including soil water movement and soil heat transfer routines from HYDRUS1D model^23^, and C and N cycle routines from the DAISY model^14^,^40^. The crop growth process were based on the PS123 generic crop model^46^, and the gross photosynthetic product was modified by water and N stress calibration factors. The N stress effect on crop growth was adopted from the CERES model.
- The numerical convergence problem of the general Richard’s equation occurs when heavy rainfall or intensive irrigation (very common in North China) happen, so a simple Green-Ampt model was used to simulate soil water infiltration and water redistribution was simulated using the Richard’s equation in the WHCNS model. In addition, to simulate root water uptake, we added a compensatory absorption mechanism to shift water uptake from dry soil zones to wetter soil zones^50^.
- The Richard’s equation is solved by the Crank-Nicolson implicit method. The convection-dispersion equations of solutes are solved by the general upwind difference method, and this technique can effectively avoid the numerical divergence and oscillation even under the conditions of dramatic changes of solute concentration and without dense nodes^39^. The initial minimum time step is set at 0.1 d, the maximum is set at 1 d, and the number of maximum iteration is set five times.
- The WHCNS model can simulate complex and intensive agricultural production systems characteristic to North China, which is typically characterized by conservation tillage, double cropping system, high planting density, film mulching, and intensive water and fertilizer inputs and other management factors. The model allows the user to study the eight irrigation methods defined by FAO56^37^ (precipitation, sprinkler, basin, border, every furrow irrigation with narrow/wide bed, alternated furrows irrigation and trickle irrigation) and the model provides four options for fertilizer application (surface, deep, mix and fertigation).
- All input and output files are stored in a user-friendly spreadsheet format, so the simulation results can be directly compared with measured data. The parameters for soil water retention curves can be input as the fitted parameters of the van Genuchten model^49^ or as water holding capacity (field capacity and wilting point)^51^. The initial concentration of soil mineral N can be input using a format of mass (mg kg^−1^) or volume (mg L^−1^).
- The model was programmed in the C++ object-oriented programming language, which will allow new features to be added in the future, such as scenario analysis and a parameter optimization module. Recently, a PEST (Parameter ESTimation) module was added to the model to facilitate the optimization of parameters^52^. In addition, a user friendly interface has been designed with the C# programming language within the Microsoft Visual Studio 2008 SDK.

### Model Calibration and Validation

The model was tested using the common data sets for comparing models presented in detail by Mirschel *et al*.^53^ collected in Muncheberg, Germany and datasets collected in the North China Plain. The data from Germany were obtained from a 6-year (1992–1998) field experiment carried out at the Centre for Agricultural Landscape and Land Use Research (ZALF) experiment station at Müncheberg, located about 40 km east of Berlin, Germany. There were three rainfed treatments with different fertilizer management in each treatment. Treatment 1 (T1) was intensively managed using inorganic fertilizer and chemical plant protection at a high level; Treatment 2 (T2) was organically managed using only organic manure and non-chemical plant protection and treatment 3 (T3) was an extensive management using a mixture of organic and inorganic fertilizers and chemical plant protection at a low level. The field management and plant and soil N measurement methods were given in detail by Mirschel *et al*.^53^. The model was calibrated using the treatment 3 datasets, and validated using the treatment 1 and 2 datasets. Calibration consisted of adjusting parameters including crop growth and N transformation in the WHCNS model by comparing the simulated and measured values of soil water content, soil nitrate concentration, crop dry matter (DM) and N uptake during the period from 3^rd^ Sept. 1992 to 27^th^ July 1998 in the T3 treatment.

The performance of the WHCNS model was compared to 14 different models (Table 1) which were tested on the Müncheberg data set at the European Conference^54^. Each of these models simulate soil water dynamic, soil N and in some cases, soil C cycles and crop growth using different theories. Some models (Expert-N, SWAP) are designed as toolkits and the users have the choice between different simulation approaches for soil water movement (balance or dynamic method), evapotranspiration and crop growth. For example, three options of crop models, CERES, SPASS, and SUCROS, were linked to Expert-N model, forming three new models denoted as ExN-CER, ExN-SPA and ExN-SUC, respectively. Two models (FASSET and CANDY) combine the capacity approach with soil pore space fractionation for simulating the movement of mobile and immobile water. Most of the models include crop modules, but model approaches range from empirical functions (NDICEA) and simplified temperature-driven approaches (SWIM, WASMOD, CANDY) to more complex mechanical models including photosynthesis, biomass partitioning and root growth development. Crop growth is represented in a generic way in some models (SWAP, Expert-N, HERMES, STAMINA, AGROTOOL, FASSET), but the others use submodels to describe specific crops (CERES, AGROSIM). Depending on their capabilities to simulate crop growth in multiple years, these models were run continuously through the whole crop rotation or were started separately for every application year (CERES, AGROSIM). Eleven models included nitrogen cycle modules, for example, AGROSIM uses a simple N balance approach and zero-order mineralization kinetics, while HERMES describes net mineralization of N from two pools using first-order kinetics. To simulate net immobilization, some models use simple C/N ratios and added organic substances (CERES, SWIM), while the others simulate C and N turnover under more complex conditions. Soil moisture and temperature are the main driving factors for C and N cycles. The denitrification process is included in all of the models except for AGROSIM, STAMINA and AGROTOOL. More detail information about the 14 soil-crop models can be found in book of ‘*Modelling water and nutrient dynamics in soil–crop systems’*^54^.

In order to test the suitability of the WHCNS model in NCP, data collected from four sites (Alxa, Alxa Left Banner in Mongolia; DBW, Dongbeiwang in Beijing, Dawenkou in Shandong province (DWK), and Quzhou in Hebei province (QZ) representing different crops (winter wheat and summer maize), soil types, climate conditions, cropping systems, and field management practices in the NCP (Table 2) were collected or compiled from the literature. The detail experimental design, field management practices and data source in each site are all listed in Table 2.

The Alxa experimental site is located in Left Banner, western Inner Mongolia, China (37.4°–41.9°N, 103.4°–106.9°E), with an elevation of 1150 m. The soils are alluvial mixed with grey desert soils. The region is classified as a warm-temperate desert arid zone with a continental climate. The average annual precipitation is 116 mm, of which 70–80% occurs during the summer season (June to September). Total annual potential evaporation reaches approximately 3005 mm, which is 20 times greater than the annual precipitation. This cropping system is a single crop of maize or wheat produced annually from the middle of April to early October. These crops comprise 80% of the cropped area. The irrigation amounts typically range from 800–1700 mm, and is typically applied through flood irrigation. Typical N fertilizer application rates are approximately 250 kg N ha^−1^. The datasets consists of three years (2005, 2008, 2009) with different water and N management for spring maize^55^.

The DBW experimental station is located in Beijing (39.5°N, 116.2°E), with an elevation of 50 m. The climate is warm-temperate continental monsoon, with an average annual air temperature of 11.5 °C. The average annual rainfall is about 560 mm with 70–80% falling from June to September. The soil is classified as a Cambisol.Surface irrigation by flooding between check banks is widely practiced in the region. The traditional cropping system is winter wheat-summer maize grown within one year. A two-year field experiment under various water and N management was conducted at this site^28^.

The DWK experimental site was located at the Shandong Agricultural University experimental station (36.0°N, 117.1°E) in Tai’an, Shandong Province, China. It is a high productivity region where the average annual grain yield for double cropping systems can approach 15,000 kg ha^−1^. The mean annual temperature is 13 °C, with the highest temperature of 26.4 °C in July and the lowest temperature of −2.6 °C in January. The mean annual rainfall was 697 mm, with the majority rainfall occurring from July to September. A winter wheat and summer maize rotation was used in the experiment, and the soil type was alluvial Cambisols. The field experiment was conducted between October 2009 and September 2012 with different tillage, cultivation, water and N treatments^34^.

The QZ experimental site was located at the China Agricultural University experimental station in Quzhou county, Hebei Province, China (36.6°N, 114.8°E). The county has a continental monsoonal climate. The annual mean air temperature is 13.1 °C. The average precipitation is 556 mm per year, and 70% of the precipitation occurs between July and September. Total potential evaporation is 1835 mm per year, three times more than annual precipitation. The soil type is alluvial Cambisols and irrigation is provided primarily from groundwater. A two-year field experiment with different water and N treatments was conducted from 1998–2000^56^.

Data from four treatments (Alxa_05_T1, DBW-04–05-T1, DWK-09–10-T1 and QZ-98–99 ) at four sites (Alxa, DBW, DWK and QZ ), respectively were chosen to calibrate the WHCNS model (Table 2). The soil hydraulic parameters for the model were taken from the literature (Table 2). Soil C and N transformation parameters came from earlier research at those sites^28^,^33^,^55^,^56^. The crop parameters were then adjusted according to the measured crop dry weight, LAI, and crop yield by the ‘trial-and-error’ method. The data from other treatments and years at the four sites were used to validate the model (Table 2).

Meteorological data, including daily maximum air temperature, minimum air temperature, air humidity, solar radiation, wind speed, and precipitation, were all collected from local weather stations.

### Estimating Model Inputs for Calibration

In order to run the model, inputs need to be estimated. We estimated the inputs for both the European dataset and the NCP dataset using the procedures described below. Model inputs were either estimated or calibrated for the calibration datasets, and then used to test the model performance using the validation datasets.

#### Soil hydraulic and solute transport parameters

Soil water retention, *θ*(*h*), and unsaturated hydraulic conductivity, *k*(*h*) functions were estimated by the van Genuchten^49^ and Mualem^57^ methods, respectively. The parameters for these functions are shown in Table 3. The hydrodynamic dispersion coefficient (*D*_*sh*_, cm^2^ d^−1^) in the liquid phase is given by^58^:


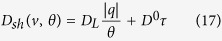


where *D*^0^ is the molecular diffusion coefficient in free water (cm^2^ d^−1^), 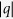 is the absolute value of the Darcian fluid flux density (cm d^−1^), and *D*_*L*_ is the longitudinal dispersivity (cm). Based on the literature^54^, the value of *D*_*L*_ was set at 3 cm, and the nitrate and ammonia values of *D*^0^ were 2.4 cm^2^ d^−1^ and 1.2 cm^2^ d^−1^, respectively. *τ* is the tortuosity factor and was estimated by Millington and Quirk^59^:


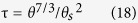


#### Soil carbon and nitrogen transformation parameters

The microbial activity in soil (soil depth) can be set in the WHCNS model, and the soil N transformation processes (mineralization-immobilization, nitrification and denitrification) are simulated based on it. In this study, the soil microbial activity was set for the top 0–30 cm in the soil. The initial parameters of N transformation were adopted from the default values of the DAISY model, and were all subsequently calibrated according to measured data. The validation parameters were obtained as following: 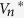, 5 g cm^−3^ d^−1^; *K*_*n*_, 50 g cm^−3^; *K*_*d*_, 0.1; *α*_d_^*^, 0.1.

The long-term dynamic simulation of SOM was not the main goal of this study, and the parameters for SOM decomposition and decay rate were all taken from the earlier studies^14^,^60^. The initial C/N ratio of residue and distribution coefficient of SOM pools are all from the literature^40^.

#### Crop parameters

The basic crop parameters were taken from Driessen and Konijon^46^. The partition coefficients and maximum photosynthetic (AMAX) were both calibrated by comparing simulated with the measured total dry weight and LAI. The calibrated crop parameters for the European dataset are shown in Table 4.

### Model performance criteria

Four statistical indices were used to evaluate the performance of each model in simulating the observed data^54^:

• The mean bias error (ME):


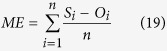


• The root mean squared error (RMSE):


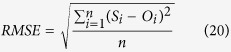


• Index of agreement (IA):


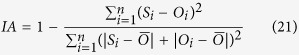


• Modeling efficiency according to Nash and Sutcliffe (NSE):


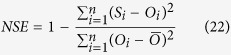


where *n* is the number of samples, *S*_*i*_ and *O*_*i*_ are the simulated and the observed values, and 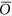 is mean of the observed data.

The *ME* presents positive and negative deviations, which makes it suitable to indicate the bias of the model error. While *RMSE* describes the average absolute deviation between simulated and observed values. The *IA* as an additional method for the evaluation of model performance and results in a range between 0 and 1. The closer *IA* is to 1, the better the simulation, similar to the coefficient of determination (*R*^2^). *NSE* compares the deviation between simulated and observed state variables with the variance of the observed values^54^.

## Results and Discussion

### Sensitivity analysis

The common datasets from Germany were used to analyze the sensitivity of all input parameters of WHCNS. The test parameters can be classified into three categories: soil hydraulic parameters (Table 3), crop parameters (Table 4), and N transformation parameters (listed in section “Soil carbon and nitrogen transformation parameters.”). A sensitivity analysis was carried out by running WHCNS with the value of a single parameter altered by 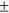
10%, while all other selected parameters and variables were constant. The affected output variables included average soil water content, soil nitrate concentration (NO_3_^−^-N), ammonia concentration (NH_4_^+^-N) in 0.9 m-soil profile, LAI, DM and crop yield.

**Figure 2:**
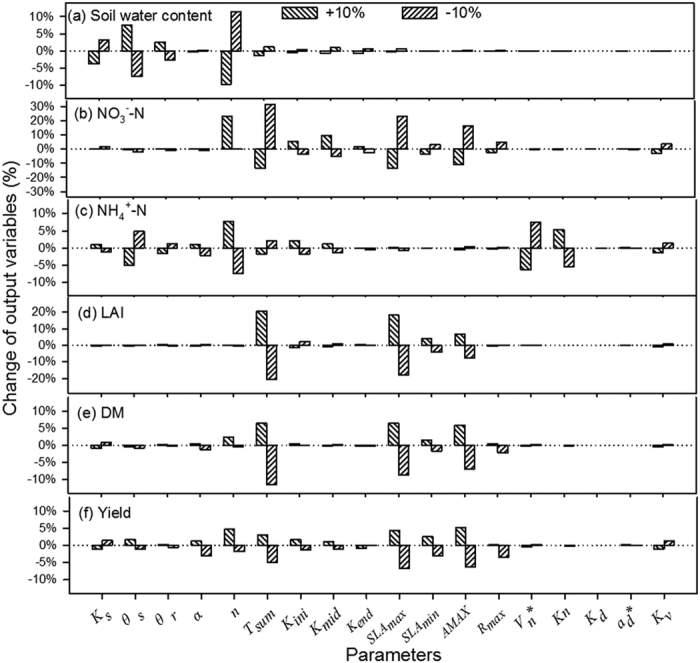
Sensitivity analysis of each parameters of WHCNS model for soil water content (**a**), NO_3_^−^-N (**b**), NH_4_^+^-N (**c**), LAI (**d**), dry matter (**e**) and yield (**f**). *K*_*s*_, saturated hydraulic conductivity; *θ*_*s*_, saturated water content; *θ*_*r*_, residual water content; *α*, the inverse of the air-entry value; *n*, pore size distribution index; *V*_n_^*^, maximum nitrification rate; *K*_*n*_, half saturation constant; *K*_*d*_, an empirical proportionality factor; *α*_d_^*^, empirical coefficient; *K*_*v*_, first order kinetic constant of volatilization; *T*_*sum*_, accumulated available temperature; *K*_ini_, *K*_mid_, *K*_end_ donote crop coefficients at initial, middle and end stages, respectively; *SLA*_*max*_and *SLA*_*min*_, denote maximum and minimum specific leaf area,respectively; *AMAX*, the maximum assimilation rate; *R*_max_, maximum root depth.

The results indicated that soil water content was more sensitive to soil hydraulic parameters than crop parameters, and the N transformation parameters had a little effect on soil water content (Fig. 2a). Among soil hydraulic parameters, *θ*_*s*_ and *n* significantly affected soil water content. Sun *et al*.^61^ found that the N transformation parameters typically had less impact on soil water content compared to soil hydraulic parameters and crop parameters. Li *et al*.^21^ also found *θ*_*s*_and *n* had a high influence on soil water content. Our results are in agreement with these findings.

Soil NO_3_^−^-N content was more sensitive to crop parameters and soil hydraulic parameters(only *n*), and the N transformation parameters had little or no effect on NO_3_^−^-N (Fig. 2b). Soil NH_4_^+^-N content was more sensitive to N transformation parameters and soil hydraulic parameters, and the crop parameters had a little inflence on NH_4_^+^-N (Fig. 2c). A change in the *n* value of van Genuchten equation, *V*_n_^*^ and *K*_*n*_ significantly affected soil NH_4_^+^-N content by over 5% (Fig. 2c), indicating that the content of NH_4_^+^-N was significantly affected by soil pore size distribution index and soil nitrification process. Crop parameters strongly affected LAI and DM, and soil hydraulic parameters and the N transformation parameters had little or no inflence on them (Fig. 2d,e). However, soil hydraulic parameters also strongly affected crop yield, but N transformation parameters had little effect on crop yield (Fig. 2f). Among the crop parameters, *T*_*sum*_, *SLA*_*max*_ and *AMAX* had the highest impact on yield. The main reason was that the value of *T*_*sum*_ determines the crop develompent stage, while *SLA*_*max*_ is directly related to the LAI. *AMAX* controls the accumulation of dry matter, and also had a closer relationship with crop yield formation. Richter *et al*.^62^ analyzed the sensitivity of crop parameters for the “Wageningen School” crop model (STAMINA), and found that the most sensitive parameters for yield were related to crop development (*T*_*sum*_ in the study) and light interception (*SLA*_*max*_ and *AMAX*).

### Performance of WHCNS model at the Müncheberg site

**Figure 3:**
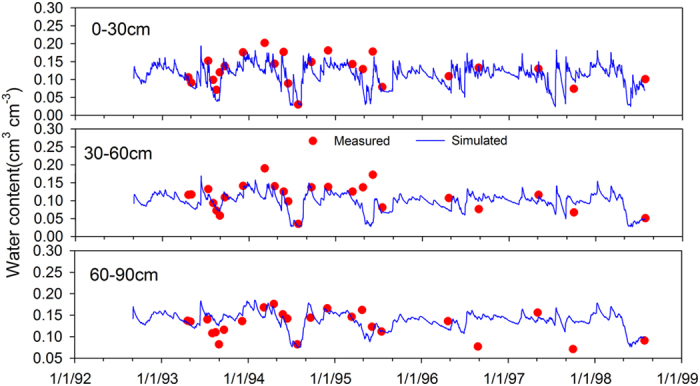
Comparison of simulated (solid lines) and measured (cycles) volumetric soil water content (cm^3^ cm^−3^) at different depths for T1 at Müncheberg site.

**Figure 4:**
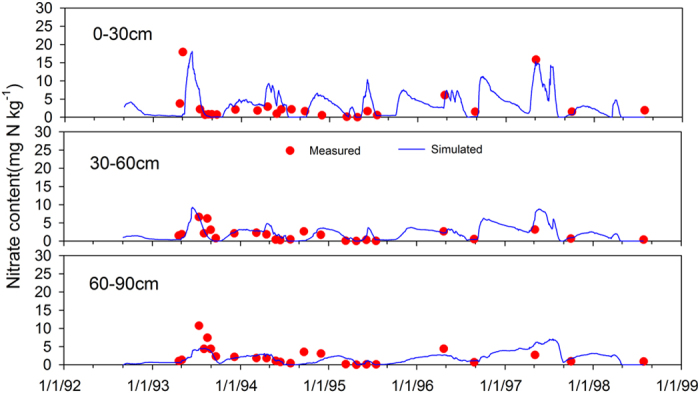
Comparison of simulated (solid lines) and measured (cycles ) soil nitrate N concentration (mg kg^−1^) at different depths for T1 at Müncheberg site.

**Figure 5:**
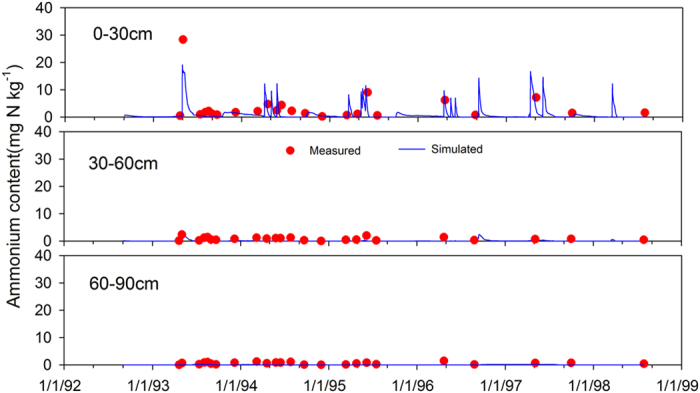
Comparison of simulated (solid lines) and measured (cycles) soil ammonium N concentration (mg kg^−1^) at different depths for T1 at Müncheberg.

**Figure 6:**
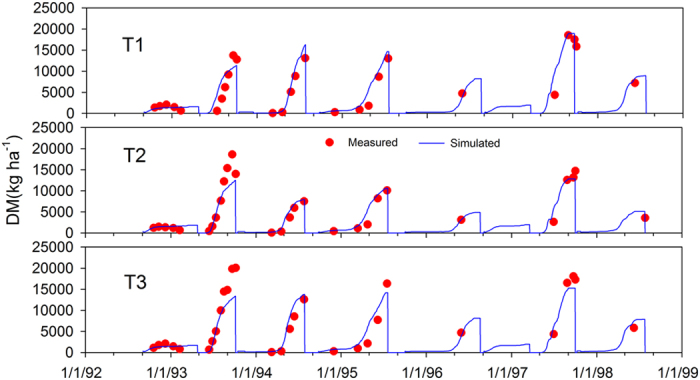
Comparison of simulated (solid lines) and measured (cycles) total dry matter for the three treatments at Müncheberg.

**Figure 7:**
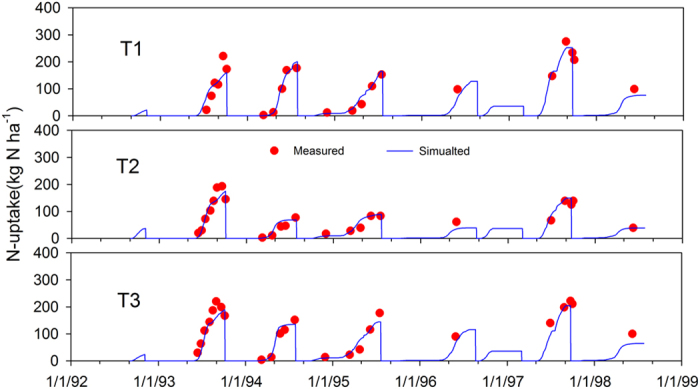
Comparison of simulated (solid lines) and measured (cycles) N-uptake by plant for three treatments at Müncheberg.

We only show the comparison results of simulated and measured soil water contents and nitrate concentrations for because of space limitations. The simulated soil water content at different soil layers in general agreed well with the measured values in T1 (Fig. 3). The comparisons of simulated and measured soil nitrate concentrations in T1 are shown in Fig. 4. The high consistency indicated the ability of the WHCNS model to simulate N transport. Figure 5 shows that the simulated peaks of ammonia (0–30 cm) agreed well with the measured values. The simulated dry matter in three treatments are shown in Fig. 6. The simulation results of all treatments were satisfactory with the exception of the yield of sugar beet in 1993, which was lower compared to measured data. The low simulated dry matter of sugar beet led to a low crop N uptake in 1993 (Fig. 7), and other simulated values matched well with the observed data. The validation results in T1 and T2 indicated that the WHCNS model could be used to simulate the water movement, fate of N and crop growth for different crop rotation systems in Germany.

The performance indices (correlation coefficient, *ME, RMSE, IA* and *NSE* ) for the WHCNS model for soil water content and nitrate concentrations at different soil layers in three plots are summarized in Table 5. There was a significant correlation between the simulated and measured. The correlation coefficient ranged from 0.331–0.694. For the soil water contents in the rooting zone (0–90 cm), the *ME* values ranged from −0.018–0.001 cm^3 ^cm^−3^, the *RMSE* values ranged from 0.026–0.031 cm^3^ cm^−3^, the *IA* values varied from 0.71–0.83, and the *NSE* values were in the range of 0.02–0.35. For soil nitrate concentration, *ME* ranged from −0.249–0.151 mg kg^−1^, the *RMSE* values varied from 1.68–2.91 mg kg^−1^, the *IA* values were in the range of 0.74–0.91, and the *NSE* values ranged from −0.18–0.65. Kersebaum *et al*.^54^ compared the simulation results of 13 soil-crop mode, found that the dynamic of mineral N usually had a negative *NSE* value (−0.81–−0.20). Given the complexity of N transformation, the low *NSE* indices are acceptable^54^.

### Comparison of WHCNS with other models at the Müncheberg site

**Figure 8:**
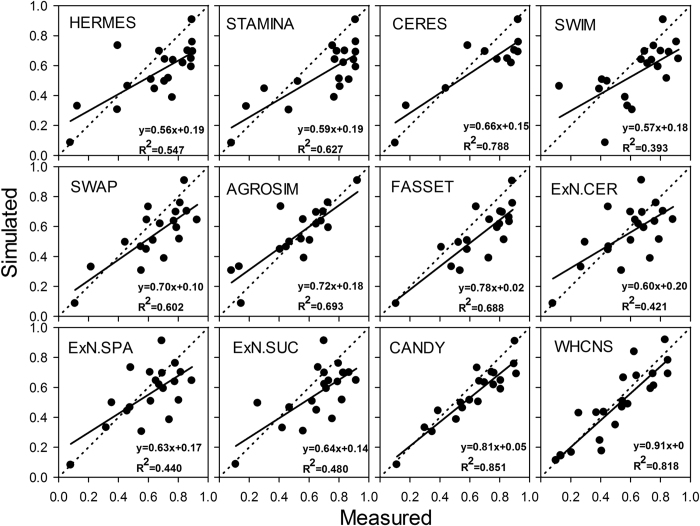
Simulated and measured gravimetric soil water contents in 0–90 cm for T1 for different models at Müncheberg (ExN.xxx = Expert-N + crop model).

**Figure 9:**
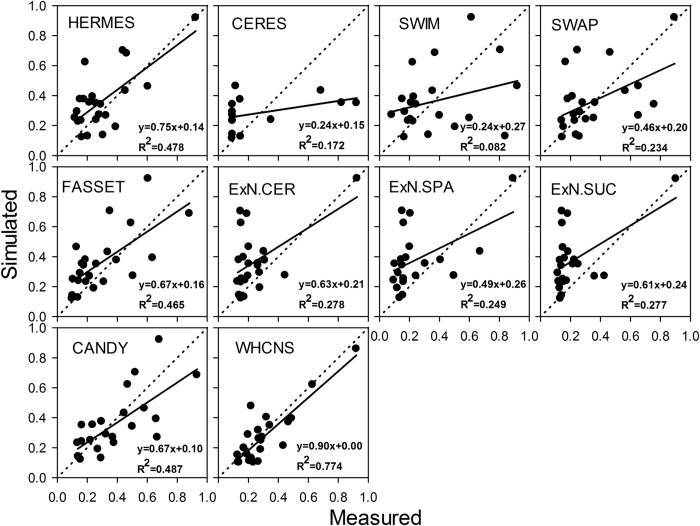
Simulated and measured soil mineral nitrogen in 0–90 cm for T1 for different models at Müncheberg (ExN.xxx = Expert-N + crop model).

**Figure 10:**
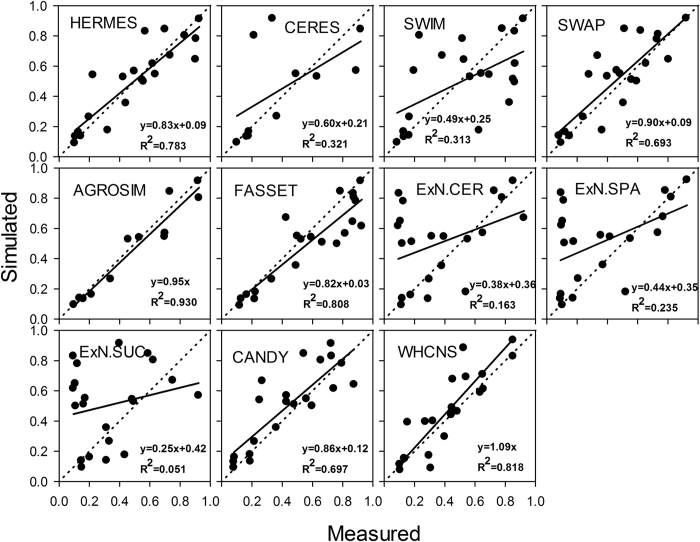
Simulated and measured N uptake by crops for T1 for different models at Müncheberg (ExN.xxx = Expert-N + crop model).

Soil water content, soil mineral N and N uptake were simulated by 12 models (some models do not simulate all processes), and the comparison results are shown in Figs 8, 9, 10 respectively. Simulated values from the WHCNS model had a high coefficient of determination for soil water content, soil mineral N and crop N uptake which were 0.818, 0.774 and 0.818, respectively, and the slopes of the fitted equation were close to 1 (soil water content, soil mineral N and crop N uptake were 0.91, 0.90 and 1.09, respectively). In addition, the four statistical indices (*ME, RMSE, IA* and *NSE*) for treatment 1 of all models are shown in Table 6. The *RMSE* values of soil water content, mineral N, dry matter and crop N uptake of the WHCNS model were relatively low among 14 compared models, and *IA* and *NSE* was higher than most of the other models. Soil mineral N and crop N uptake simulated by WHCNS model had the relatively high *NSE* value in all simulations (0.38 and 0.79, respectively) compared with the other models. The *IA* values of dry matter, crop N uptake and soil water content of 0.96, 0.93 and 0.87, respectively, also indicated good model performance. Overall, the WHCNS model gave good agreement with measured data for simulating the dynamics of soil water content and nitrate concentrations in soil profile, as well as crop dry matter and crop N uptake.

There are many reasons that lead to the difference in simulations of soil water movement and N transport. The different approaches for simulating soil water balance (balance or dynamic method) led to differences in simulated soil water content. Krobel *et al*.^63^ compared the performances of the DNDC (balance method) and DAISY (Richard’s equation) models in simulating soil water movement and found that the DAISY model had higher accuracy than the DNDC model in simulating soil water content. Crop water uptake is also influenced by root dynamics. Models that simulate more detailed root water uptake generally perform better in simulating soil water content than those with lesser detail^50^,^64^. Because the compensated root water method was adopted in the WHCHS model, it performed better than most of the other models in simulating soil water content. For N transport, the complexity of N transformations considered in the model affects the simulated result of soil mineral N content. Eleven of the models included N cycle process modules^54^. AGROSIM uses a simple N balance approach and zero-order mineralization kinetics, while HERMES describes net mineralization of N from two pools using first-order kinetics. For net immobilization, some models use simple C/N ratios and added organic substances (CERES, SWIM), while others simulate C and N turnover under more complex conditions (such as soil moisture and temperature as the main driving factors for C and N cycles)^54^. In additional, those influencing factors of soil water movement and crop growth will also affect N transformation and transport. In this study, the integrated model (WHCNS) combined the best aspects of widely used soil-crop models, and improved the ability of model to represent various environmental conditions, and gave better performance compared to most of the other 14 soil–crop system models.

### Calibration and evaluation of WHCNS in North China

#### Soil water and nitrogen

**Figure 11:**
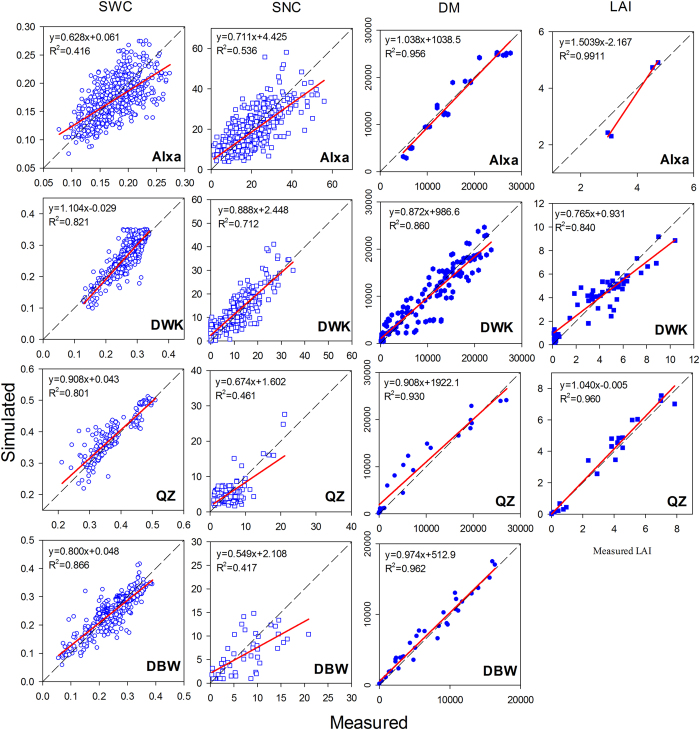
The relationship between simulated and measured soil volumetric water content (SWC, ‘
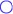
’,cm3 cm^−3^), soil nitrate concentration (SNC, ‘
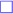
’, kg ha^-1^), crop dry mass (DM,‘
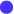
’, kg ha^−1^) and leaf area index (LAI, ‘
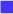
’) for WHCNS model in North China.

Figure 11 shows the validation results of soil water content, nitrate concentrations, dry matter and LAI in four sites in the NCP. The coefficients of determination between simulated and measured soil water content and nitrate concentrations are in the range of 0.42–0.87 and 0.42–0.71, respectively at four sites. For the validation of soil water content, the *RMSE* values ranged from 0.03–0.04 cm^3^ cm^−3^ (Table 7); the *IA* indices were all over 0.78; and the *NSE* values had the lowest value of 0.34 in Alxa, and values of 0.78, 0.76 and 0.63 in DWK, QZ and DBW, respectively. Hu *et al*.^29^ applied the RZWQM2 model to simulate soil water content for double cropping system in NCP, and the *RMSE* range was 0.03–0.07 cm^3 ^cm^−3^. Van Liew and Garbrecht^65^ analyzed several soil water models and suggested that the simulation performed well when the value of NSE > 0.36 and IA > 0.7. In this study, these statistical indices are all within these reported acceptable ranges.

The validation results for the nitrate simulation in different soil layers showed that all the *IA* indexes were more than 0.77, the *RMSE* values ranged from 2.58–7.04 mg kg^−1^, the *ME* indices ranged between −1.23 and −0.28 mg kg^−1^, which showed the model underestimated the soil nitrate content. The *NSE* values ranged from 0.29–0.63 for the four sites. This might be due to the complex N transformation process. Kersebaum *et al*.^54^ compared the simulation results of 13 soil-crop models, and found that the dynamic of mineral N usually had a negative NSE value (−0.81–−0.20). Hu *et al*.^29^ reported that the value of NSE in general was negative, which was caused by the high variability observed for soil NO_3_^−^-N measurement. Given the complexity of N transformation, the ranges of these indices appear to be acceptable. These results indicated that the model performed well in simulating soil water dynamic and nitrate transport under different water and N management practices.

#### Crop growth

**Figure 12:**
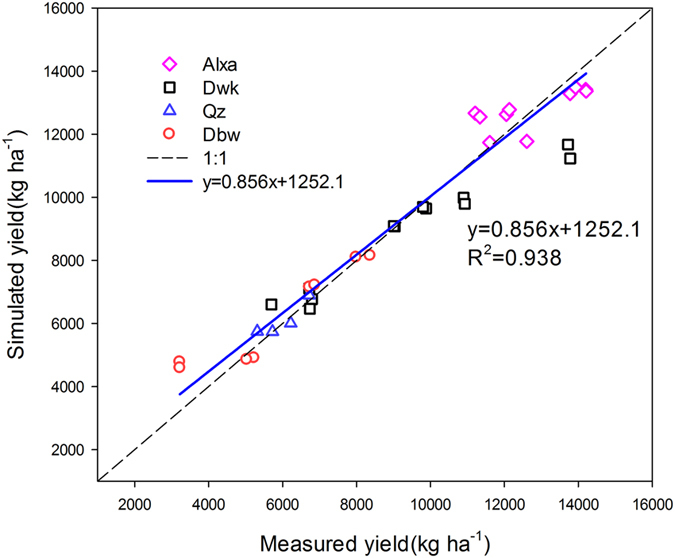
The relationship between simulated and measured crop yield in four sites for the WHCNS model in North China.

The species of crop growth simulated at the four sites included winter wheat, summer maize and spring maize. The validation results showed that the model explained more than 84% of the variation in LAI and dry matter (Fig. 11), and all the slopes of the regression lines were close to 1, except for the LAI in Alxa (1.5) due to limited measured data (only four measurements), while the *RMSE* values for dry matter ranged from 1006–2962 kg ha^−1^ (Table 7). Total dry matter had the largest error in QZ (2962 kg ha^−1^), and the smallest error in DWK (1006 kg ha^−1^). Figure 12 shows the simulation results of crop yields at the four sites for the validation datasets. In general, the simulation results matched well with observed values. The determination coefficients for the four sites was 0.94 (Fig. 12). The *RMSE* values ranged from 243–1097 kg ha^−1^, and the *IA* indices were all over 0.70 (Table 7). Palosuo *et al*.^66^ compared the performance of eight widely used soil-crop system models for winter wheat yield simulation at seven sites in Northern and Central Europe, and found that the DAISY and DSSAT models gave better performance, the values of *RMSE* were the lowest (1428 and 1603 kg ha^−1^, respectively) and *IA* (0.71 and 0.74, respectively) was the highest. Among those models, CROPSYST underestimated crop yields (*ME*, −1186 kg ha^−1^), while HERMES, STICS and WOFOFST overestimated crop yields with *ME* values of 1174, 1272 and 1213 kg ha^−1^, respectively. In another comparison of nine crop models for spring barley yield simulation at seven sites in Northern and Central Europe, Rotter *et al*.^67^ found that HERMES, MONICA and WOFOST outperformed other models in simulating crop yield with the lowest *RMSE* values of 1124, 1282 and 1325 (kg ha^−1^), respectively. Compared to those indices simulated by these models, the WHCNS performed reasonably well in simulating crop yield in North China.

**Figure 13:**
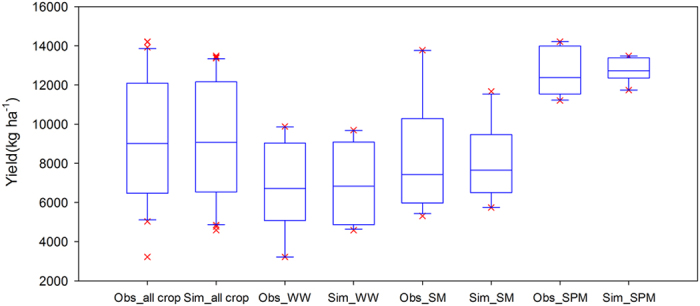
Distribution of simulated and measured yields for different crops for the WHCNS model in North China (Obs, Observed; Sim, Simulated; WW, Winter Wheat; SM, Summer Maize; SPM, Spring Maize).

Figure 13 shows the box-plot for the simulated and measured crop yields of winter wheat, summer maize and spring maize. The results showed that the data distribution of the simulated total and specific crop yields was similar to that of the measured ones but with less variance. This issue was also found by many researchers when simulating the crop growth at a regional scale^68^,^69^. This was probably due to lower sensitivity of the model to the N fertilizer application because there are large amount of accumulated mineral N in soil profile in North China^28^,^34^,^56^,^70^. Another reason may be the uncertainty of the observed yield caused by the extreme climate, diseases and pests^66^,^67^. In our study, the extreme climate led to an abnormally low yield of winter wheat in 2011 (3220 kg ha^−1^) in DBW. However, the current models do not include modules to simulate impacts of these factors and further study is needed.

The WHCNS model successfully simulated soil water content, nitrate concentrations, crop yields, and LAI in the North China, and explained the difference in crop yield under different water and N management practices. Hence, the model has the potential to be used in simulating soil water movement, N cycle, and crop development under different climate conditions, soils, crop rotations and a variety of water and fertilizer management practices in NCP.

However, as a newly developed model, more testing is needed using long-term datasets from different sites to assess the uncertainty caused by climate change. In terms of functions, the new model cannot simulate the effects of other nutrients (such as phosphorus fertilizer), diseases and pests on crop growth. In addition, the greenhouse gas (CH_4_), dissolved carbon (DOC) and the dissolved N (DON) play an important role in lowland agricultural production. The WHCNS model cannot currently simulate these processes. In order to simulate hydrologic processes on a regional scale, a hydrological and groundwater module should be incorporated in the model in the future.

## Conclusion

The open access dataset from a field experiment in Germany, available after a modeling workshop in Europe was used to test the newly developed WHCNS model. We then compared the results with the simulation results of 14 other soil-crop system models. The WHCNS model ranked in the top three based on the *NSE* and *IA* indices for soil water contents (*NSE*, 0.36; *IA*, 0.87), soil nitrate concentrations (*NSE*, 0.38; *IA*, 0.79), crop dry matter (*NSE*, 0.84; *IA*, 0.96) and crop N uptake (*NSE*, 0.36; *IA*, 0.87). We concluded that WHCNS gave good performance in simulating soil water dynamic, nitrate and ammonia transport, crop growth development and grain yield under different crop rotation and fertilizer management practices.

In addition, the WHCNS model was also tested using datasets from four different sites in North China across different soils, climate conditions, cropping systems, and intensive water and fertilizer management practices. The *IA* and *NSE* values for LAI and dry matter were close to 1. The determination coefficient for the crop yields ranged from 0.84–0.99. And the *RMSE* values ranged from 243–1097 kg ha^−1^. The *IA* indices were all over 0.70. All these results indicated that the newly developed WHCNS can be used to analyze and evaluate the grain yield, fate of N, WUE and NUE in the intensively cultivated farmland in North China.

## Additional Information

**How to cite this article**: Liang, H. *et al*. An integrated soil-crop system model for water and nitrogen management in North China. *Sci. Rep.* **6**, 25755; doi: 10.1038/srep25755 (2016).

## Figures and Tables

**Table 1:** Comparison of 15 crop-soil models.

Model name	Timeline	Timestep	Scale	Type	Simulates†
STAMINA	Discon.	Hour-Day	Field	1D	w,p
AGROSIM	Cont.	Day	Field	1D	w,p
AGROTOOL	Discon.	Day	Field	1D	w,p
NDICEA	Discon.	Week	Field	1D	w,n,c
SWAP/ANIMO	Cont.	Day	Field	1D	w,n,c,p
SWIM	Cont.	Day	River basin	2D	w,n,c,p
HERMES	Cont.	Day	Field-meso	1D	w,n,p
WASMOD	Discon.	Day	Catchment	2D	w,n,c
CERES	Cont.	Day	Field	1D	w,n,p
ExN-CER	Cont.	Day	Field	1D	w,n,c,p
ExN-SPA	Cont.	Day	Field	1D	w,n,c,p
ExN-SUC	Cont.	Day	Field	1D	w,n,c,p
FASSET	Cont.	Day	Farm	1D	w,n,c,p
CANDY	Cont.	Day	Field	1D	w,n,c
WHCNS	Cont.	Day	Field	1D	w,n,c,p

Note: ExN-CER, ExN-SPA and ExN-SUC are the linking of Expert-N model with the crop model options of CERES, SPASS and SUCROS, respectively.

Cont: continuous; discontinuous; opt: optional.

^†^Model simulates – w: water; n:nitrogen; c: carbon cycle; p: plant growth.

**Table 2:** Datasets used for model calibration and validation in North China.

ID	Location	Rotation	Years	Treatments	Crop	Irr	Fer	Data†	Group‡	Sources
Alxa	104.5°E, 39.5°N	SPM	20052008–2009	Alxa-05-T1	SPM	825	179	wndly	C	Hu *et al*.^54^
				Alxa-05-T2	SPM	630	317	wndly	V	
				Alxa-T1	SPM	750	138	wndy	V	
				Alxa-T2	SPM	750	92(150)	wndy	V	
				Alxa-T3	SPM	570	138	wndy	V	
				Alxa-T4	SPM	570	92(114)	wndy	V	
DBW	116.2°E, 39.5°N	WW- SM	2004–2006	DBW-04-05-T1	WW	335	300	wndy	C	Wang *et al*.^28^
				DBW-05-T1	SM	100	250	wndy	C	
				DBW-05–06-T1	WW	335	300	wndy	V	
				DBW-06-T1	SM	50	250	wndy	V	
				DBW-04-05T2	WW	305	190	wndy	V	
				DBW-05-T2	SM	100	75	wndy	V	
				DBW-05–06-T2	WW	265	135	wndy	V	
				DBW-06-T2	SM	50	140	wndy	V	
DWK	117.1°E, 36.0°N	WW- SM	2009–2011	DWK-09–10-T1	WW	375	315	wndly	C	Li *et al*.^34^
				DWK-10-T1	SM	75	225	wndly	C	
				DWK-10–11-T1	WW	300	315	wndly	C	
				DWK-11-T1	SM	75	225	wndly	C	
				DWK-09–10-T2	WW	300	210	wndly	V	
				DWK-10-T2	SM	75	160	wndly	V	
				DWK-10–11-T2	WW	225	210	wndly	V	
				DWK-11-T2	SM	75	160	wndly	V	
				DWK-09–10-T3	WW	300	360	wndly	V	
				DWK-10-T3	SM	75	450	wndly	V	
QZ	114.8°E, 36.6°N	WW- SM	1998–2000	DWK-10–11-T3	WW	225	390	wndly	V	Hu *et al*.^55^
				DWK-11-T3	SM	75	450	wndly	V	
				QZ-98–99	WW	296	259	wndly	C	

Note: SPM, Spring Maize; SM, Summer Maize, WW, Winter Wheat; Irr, Irrigation (mm); Fer, Fertilizer (kg N ha^–1^), the values in the brackets are the nitrogen amounts in irrigation water.

^†^Data available: w, soil water content; n, soil nitrate concentration; d, crop dry matter; l, leaf area index; y, yield.

^‡^C and V represented for calibration data and validation data in application, respectively.

**Table 3:** Soil physical and hydraulic properties for soil profiles of the three experiments in Muncheberg^53^.

Plot	Soil Layer (cm)	BD(g cm^−3^)	Particle fraction (%)			Texture (USDA)	*θ*_*r*_(cm^3^ cm^−3^)	*θ*_*s*_(cm^3^ cm^−3^)	*α*(1 cm^−1^)	*n*	K_s_(cm d^−1^)
			Sand	Silt	Clay						
Plot 1	0–30	1.45	90	3	7	Sand	0.027	0.385	0.021	2.013	92
	30–60	1.5	90	5	5	Sand	0.027	0.319	0.027	2.179	162
	60–90	1.55	80	8	12	Sandy loam	0.065	0.385	0.028	2.147	30
	90–120	–	90	6	4	Sand	0.027	0.319	0.027	2.379	162
	120–150	–	90	7	3	Sand	0.027	0.319	0.027	2.379	162
	150–225	–	90	8	2	Sand	0.027	0.319	0.027	2.379	162
Plot 2	0–30	1.45	85	10	5	Loamy sand	0.027	0.385	0.021	2.013	92
	30–90	1.5	90	5	5	Sand	0.027	0.319	0.027	2.179	162
	90–130	1.55	80	8	12	Sandy loam	0.065	0.385	0.028	2.147	30
	130–170	–	80	10	10	Sandy loam	0.027	0.302	0.028	2.147	30
	170–180	–	90	5	5	Sand	0.027	0.319	0.027	2.379	162
	180–225	–	90	5	5	Sand	0.027	0.319	0.027	2.379	162
Plot 3	0–30	1.45	85	9	6	Loamy sand	0.027	0.385	0.021	2.013	92
	30–100	1.5	90	5	5	Sand	0.027	0.319	0.027	2.379	162
	100–110	1.55	81	6	13	Loamy sand	0.065	0.385	0.028	2.147	30
	110–225	–	80	9	11	Sandy loam	0.065	0.385	0.028	2.147	30

Note: BD is bulk density; *θ*_*r*_ is the residual water content; *θ*_*s*_ is the saturated water content; α is the inverse of the air-entry value; *n* is a pore size distribution index; *K*_*s*_ is the saturated hydraulic conductivity (*l* = 0.5).

**Table 4:** Calibrated crop parameters used in the WHCNS model for the European dataset.

Parameters	Description	Crops			
		Sugarbeet	WinterWheat	Winterbarley	Winterrye
*Tbase*	Base temperature (°C)	0	0	0	0
*T*_*sum*_	Accumulated temperature (°C)	2100	2110	2000	1800
*Ke*	Extinction coefficient	0.6	0.6	0.6	0.44
*K*_*ini*_	Crop coefficient in initial stage	0.6	0.6	0.6	0.6
*K*_*mid*_	Crop coefficient in middle stage	1.35	1.35	1.35	1.35
*K*_*end*_	Crop coefficient in end stage	0.6	0.6	0.6	0.6
*SLA*_*max*_	The maximum specific leaf area (m^2^ kg^−1^)	28	22	25	22
*SLA*_*min*_	The minimum specific leaf area (m^2^ kg^−1^)	18	14	18	15
*AMAX*	The maximum assimilation rate (kg ha^−1^ h^−1^)	45	55	45	45
*R*_*max*_	Maximum root depth (cm)	90	90	90	90

**Table 5:** Statistical indices for simulated soil water content and nitrate concentration at different layers for three treatments for the WHCNS model.

Items	Soil layers	*y* = a*x* + b	*R*^*2*^	*ME*	*RMSE*	*IA*	*NSE*
Soil water content(cm^3^ cm^−3^)	0–30 cm	y = 0.793x + 0.040	0.613*	−0.018	0.031	0.83	0.35
	30–60 cm	y = 0.78x + 0.036	0.652*	−0.016	0.030	0.74	0.14
	60–90 cm	y = 0.512x + 0.066	0.245*	0.001	0.026	0.71	0.02
Soil nitrate concentration(mg N kg^−1^)	0–30 cm	y = 0.860x + 0.784	0.694*	−0.177	2.91	0.91	0.65
	30–60 cm	y = 0.838x + 0.509	0.416*	−0.249	1.68	0.78	0.39
	60–90 cm	y = 0.740x + 0.674	0.331*	0.151	1.95	0.74	−0.18

Note: *denotes a significant level (p < 0.05).

**Table 6:** Summary of model performance of participating models for water, soil mineral N, dry matter and crop N uptake in treatment 1.

Models	Soil water content				Soil mineral N			
	*ME*	*RMSE*	*IA*	*NSE*	*ME*	*RMSE*	*IA*	*NSE*
NDICEA	–	–	–	–	−6.3	23.2	0.81	0.43
STAMINA	2.6	18.7	0.64	0.23	–	–	–	–
AGROSIM	39.0	44.9	0.50	−3.11	–	–	–	–
SWAP	1.8	19.3	0.79	0.26	−19.9	34.1	0.61	−0.20
SWIM	7.1	23.8	0.65	−0.22	−0.4	41.6	0.56	−0.81
HERMES	4.1	24.0	0.80	−0.22	−11.1	25.8	0.80	0.32
CERES	−4.1	14.2	0.93	0.66	−14.2	21.6	0.59	−0.65
ExN-CER	12.0	25.0	0.74	−0.33	−23.4	35.7	0.66	−0.34
ExN-SPA	10.6	24.8	0.76	−0.31	−26.2	38.0	0.63	−0.51
ExN-SUC	16.7	26.2	0.70	−0.45	−26.3	37.8	0.65	−0.49
FASSET	3.9	19.5	0.82	0.02	6.9	35.1	0.76	−0.29
CANDY	2.8	23.3	0.79	−0.13	1.6	24.5	0.83	0.39
WHCNS	9.4	18.2	0.87	0.36	−13.9	22.3	0.79	0.38
	Dry matter				Crop N uptake			
	*ME*	*RMSE*	*IA*	*NSE*	*ME*	*RMSE*	*IA*	*NSE*
NDICEA	–	–	–	–	–	–	–	–
STAMINA	−1,366	2,766	0.78	0.49	–	–	–	–
AGROSIM	107	775	0.99	0.96	17.4	37.6	0.92	0.63
SWAP	–	–	–	–	−5.0	33.8	0.89	0.61
SWIM	–	–	–	–	−18.3	56.6	0.67	−0.14
HERMES	214	1,403	0.96	0.87	7.0	36.9	0.90	0.54
CERES	−1,266	1,884	0.96	0.85	−16.2	60.8	0.71	0.07
ExN-CER	−844	1,871	0.93	0.75	−18.6	69.0	0.61	−0.71
ExN-SPA	−1,246	2,232	0.89	0.64	−18.3	68.1	0.66	−0.66
ExN-SUC	449	2,619	0.89	0.49	−15.3	77.3	0.50	−1.14
FASSET	455	3,327	0.80	0.19	2.3	25.2	0.94	0.80
CANDY	–	–	–	–	11.5	51.8	0.83	0.05
WHCNS	1,151	2,217	0.96	0.84	−11.8	35.5	0.93	0.79

Note: ExN-CER, ExN-SPA and ExN-SUC are the linking of Expert-N model with the crop model options of CERES, SPASS and SUCROS, respectively.

**Table 7:** Summary of the WHCNS model performance for water, soil nitrate, crop dry matter, LAI and yield at four sites.

Location	Indices	Calibration					Validation				
		SWC	SNC	DM	LAI	Y	SWC	SNC	DM	LAI	Y
Alxa	*n*	155	95	6	2	1	718	419	24	2	9
	*ME*	0.01	−0.60	−1608	0.30	131	−0.01	−1.14	−478	0.17	168
	*RMSE*	0.03	7.70	1646	0.56	131	0.03	6.87	1444	0.40	870
	*IA*	0.76	0.90	0.94	0.93	–	0.78	0.87	0.99	0.96	0.70
	*NSE*	0.35	0.47	0.78	0.81	–	0.34	0.54	0.95	0.88	0.49
DWK	*n*	372	77	31	19	4	1011	232	93	38	8
	*ME*	−0.02	−1.17	301	−0.20	−199	−0.01	−1.23	333	0.11	−489
	*RMSE*	0.04	6.84	1032	0.85	870	0.04	7.04	1006	1.13	1097
	*IA*	0.87	0.95	0.99	0.96	0.96	0.95	0.90	0.99	0.94	0.94
	*NSE*	0.75	0.68	0.95	0.79	0.89	0.78	0.63	0.96	0.74	0.82
QZ	*n*	72	41	6	6	1	216	124	17	15	3
	*ME*	0.01	0.50	2030	0.02	175	0.01	−0.28	1373	0.17	102
	*RMSE*	0.03	3.09	3202	0.54	175	0.03	2.58	2962	0.53	243
	*IA*	0.89	0.92	0.96	0.97	–	0.94	0.88	0.97	0.99	0.93
	*NSE*	0.71	0.66	0.86	0.85	–	0.76	0.49	0.89	0.95	0.76
DBW	*n*	117	12	8	–	2	356	35	26	–	6
	*ME*	−0.01	−0.51	453	–	181	0.03	−1.12	296	–	38
	*RMSE*	0.04	3.28	656	–	280	0.04	4.18	1091	–	262
	*IA*	0.91	0.82	0.99	–	0.95	0.89	0.77	0.99	–	0.95
	*NSE*	0.72	0.43	0.96	–	0.76	0.63	0.29	0.96	–	0.88

Note: *n* is the number of samples; SWC, soil water content (cm^3^ cm^−3^); SNC, soil nitrate concentration (mg kg^−1^); DM, crop dry matter (kg ha^−1^); Y, Yield (kg ha^−1^).
